# Surfactant-Free Formate/O_2_ Biofuel Cell with Electropolymerized Phenothiazine Derivative-Modified Enzymatic Bioanode

**DOI:** 10.1021/acsabm.3c00502

**Published:** 2023-09-26

**Authors:** Motohiro Kosugi, Ryoichi Tatara, Yuki Fujii, Shinichi Komaba

**Affiliations:** Department of Applied Chemistry, Tokyo University of Science, Shinjuku, Tokyo 162-8601, Japan

**Keywords:** biofuel cell, formate, electropolymerization, phenothiazine, carbon

## Abstract

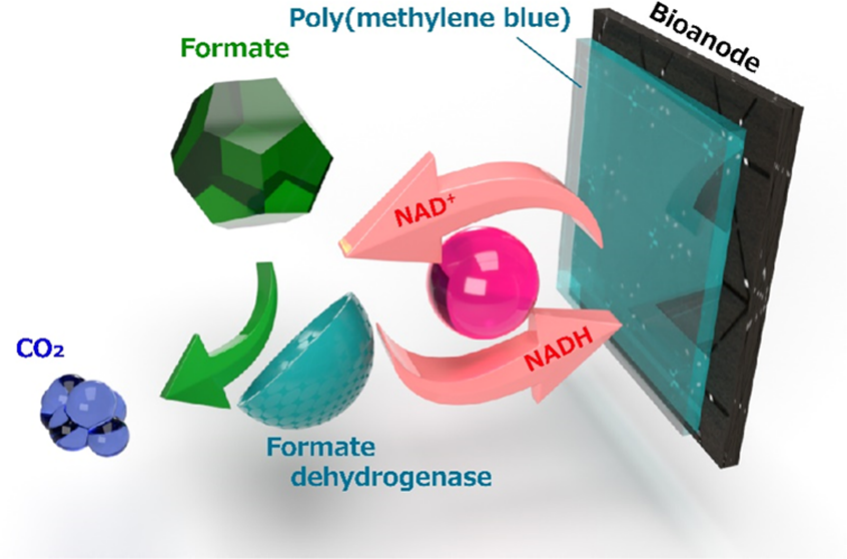

A formate (HCOO^–^) bioanode was developed by utilizing a phenothiazine-based electropolymerized layer deposited on sucrose-derived carbon. The electrode modified with NAD-dependent formate dehydrogenase and the electropolymerized layer synergistically catalyzed the oxidation of the coenzyme (NADH) and fuel (HCOO^–^) to achieve efficient electron transfer. Further, the replacement of carbon nanotubes with water-dispersible sucrose-derived carbon used as the electrode base allowed the fabrication of a surfactant-free bioanode delivering a maximum current density of 1.96 mA cm^–2^ in the fuel solution. Finally, a separator- and surfactant-free HCOO^–^/O_2_ biofuel cell featuring the above bioanode and a gas-diffusion biocathode modified with bilirubin oxidase and 2,2′-azino-bis(3-ethylbenzothiazoline-6-sulfonate) was fabricated, delivering a maximum power density of 70 μW cm^–2^ (at 0.24 V) and an open-circuit voltage of 0.59 V. Thus, this study demonstrates the potential of formic acid as a fuel and possibilities for the application of carbon materials in bioanodes.

## Introduction

Enzymatic biofuel cells generate electricity through the enzyme-catalyzed oxidation of organic materials^1−8^ such as saccharides (glucose,^9−13^ fructose,^14,15^ maltose,^16^ and starch^17−19^), alcohols (methanol^20−23^ and ethanol^24−29^), and formic acid^30,31^ and feature milder operation conditions (room temperature, neutral pH, and ambient pressure) compared to typical inorganic-catalyst fuel cells. The substrate specificity of enzymes obviates the need for anode–cathode separation and thus facilitates cell miniaturization and applications in wearable^32−37^ and implantable^38−41^ devices. Among the numerous bioanode fuels, formic acid exhibits relatively low redox potential, and the corresponding biofuel cells therefore feature high theoretical voltage.^30,31,42^ Formic acid is a liquid under ambient conditions and can be easily stored and transported. Furthermore, formic acid is the first stable compound produced by the reduction of carbon dioxide, which makes formic acid fuel cells an attractive part of the energy cycle based on carbon dioxide reduction technologies.^31^

In view of their general applicability to a wide variety of fuels, nicotinamide adenine dinucleotide (NAD)-dependent enzymes are often used to catalyze bioanodic oxidation reactions^9,20−25,28,29,34,43^ and even allow the realization of multistep bioanodic enzymatic reactions mimicking in vivo metabolic reactions.^20,23−25^ Given that oxidation catalyzed by NAD-dependent enzymes is accompanied by the reduction of the NAD coenzyme (eq 1), electron transfer from the fuel to the bioanode inevitably involves the oxidation of NADH as follows
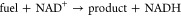
1

However, efficient electron transfer requires efficient electrocatalysis because of the high overpotential of NADH oxidation.^9,20−25,28,29,34,43^ Kano et al. reported formate/O_2_ biofuel cell system utilized with formate dehydrogenase,^31^ and Zhu et al. reported a high-voltage formate/O_2_ biofuel cell utilizing gold nanoparticles as catalysts,^30^ while Minteer and Banta et al. achieved the multistep oxidation of methanol on an electrode modified with electropolymerized methylene green^20^ and methylene blue.^44^ Gorton et al. reported poly phenothiazine derivative-based electropolymerized film is effective for electrocatalytic oxidation of NADH.^45^ However, to date, optimized design strategies, including those using electrocatalysts to fabricate enzymatic bioanodes for formic acid oxidation, have not been reported.

Carbon materials are widely used in enzymatic biofuel cells to increase the enzyme modification density.^43,45−51^ In particular, carbon nanotubes (CNTs) enjoy high popularity due to their high specific surface area and electrical conductivity.^13,16,18,51−53^ The high propensity of CNTs for agglomeration is typically mitigated through the addition of surfactants to disperse CNT for drop-coat process.^16,18,49,51,52^ However, they inevitably dissolve from the CNT electrode into the electrolyte and thus hydrophilize an opposite gas-diffusion biocathode to cause electrolyte leakage and/or reduce fuel-cell performance due to electrolyte flooding and the loss of oxygen supply to the biocathode.^49^ Therefore, the preparation of surfactant-free bioanodes is essential for the realization of high-performance formate/O_2_ biofuel cells.

To address the above-mentioned bottlenecks, we herein fabricated and tested a formate/O_2_ biofuel cell with a gas-diffusion biocathode and a surfactant-free bioanode. Cost-effective redox-active phenothiazine derivatives used as electropolymerization monomers were tested as bioanode electrocatalysts,^54−59^ and sucrose-derived carbon (SCR) previously developed for a negative electrode material for sodium-ion batteries^60^ was uniquely used as the bioanode support, as this material (particularly when synthesized at low temperatures) is well dispersible in water.

## Experimental Section

### Materials

Formate dehydrogenase from *Candida boidinii* (FDH; 0.4 U mg^–1^, Sigma-Aldrich Co.) and bilirubin oxidase from *Myrothecium verrucaria* (BOD; 2.45 U mg^–1^, Amano Enzyme) were used as biocatalysts. 9,10-Phenanthrenequinone (PQ; Sigma-Aldrich Co.), methylene blue (MB; Sigma-Aldrich Co.), thionin acetate (TH; Sigma-Aldrich Co.), and Azure A chloride (AA; Sigma-Aldrich Co.) were used as bioanode electrocatalysts. *N*,*N*-Dimethylformamide (DMF, ≥99.0%; Nacalai Tesque, Inc., Japan) was used as a solvent for PQ. An aqueous KNO_3_ (Wako Chemical) solution was used for electropolymerization. 2,2′-Azino-bis(3-ethylbenzothiazoline-6-sulfonic acid) (ABTS; Sigma-Aldrich Co.) was used as a biocathode mediator. Glutaraldehyde (GA; 50% solution in water, Kanto Chemical) and poly(ethylene glycol) diglycidyl ether (PEGDGE; *M*_*n*_ = 500 Da, Sigma-Aldrich Co.) were used as bioanode cross-linkers. Triton X-100 (TX; Nacalai Tesque, Inc., Japan) was used as a surfactant for dispersing CNT in water. Sodium poly-γ-glutamate (PGluNa; Sigma-Aldrich) and styrene–butadiene rubber (SBR; 48.4 wt % latex form, TRD2001, JSR Co.) were used as binders. Sodium formate (≥99%, Sigma-Aldrich Co.) was used as the fuel, and sucrose (Nacalai Tesque) was used as the carbon source. Multiwalled CNTs (6–13 nm × 2.5–20 μm, Sigma-Aldrich Co.), Ketjen black (KB; EC600JD, Lion Specialty Chemicals), and acetylene black (AB, 50% compressed, Strem Chemicals) were used as conductive agents. A carbon fiber material consisting of an unwoven carbon fabric (2 mm thick carbon felt (CF), Tsukuba Materials Information Laboratory, Ltd., Japan) was employed to support the bioanode, while carbon paper (CP; TGP-H-120, Toray) was used to support the biocathode. A stainless-steel mesh (100 mesh, Tokyo Screen Co., Ltd.) was used as a current collector.

Phosphate-buffered solutions (PBS) were prepared by dissolving KH_2_PO_4_ (Nacalai Tesque, Inc.) and Na_2_HPO_4_ (Nacalai Tesque, Inc.) in water, and solution pH was adjusted using a pH meter. Deionized (DI) water with a conductivity of <1.0 μS cm^–1^ was obtained using a purification system (Purelite, PRA-0015, Organo) and used in all experiments. The above chemicals and enzymes were used as received without further purification.

### GCE/CNT/SBR/TX/(polyMB, polyTH, or polyAA) Electrodes

An aqueous CNT dispersion (10 μL) containing the dispersant (TX) and binder (SBR) (CNT: SBR: 0.5% TX solution = 1 mg: 0.53 mg: 0.5 mL) was drop-coated on a glassy carbon electrode (GCE, 5 mm diameter). The resulting electrode was then immersed into a 1 mM solution of the desired monomer in [0.1 M KNO_3_ + 0.1 M PBS] (pH 7.0) and subjected to cyclic voltammetry to induce the deposition of the electropolymerized films.

### CF/CNT/SBR/TX/(polyMB, polyTH, or polyAA)/FDH Bioanodes

An aqueous CNT dispersion (180 μL) containing the dispersant (TX) and binder (SBR) (CNT: SBR: 0.5% TX solution = 1 mg: 0.53 mg: 0.5 mL) was drop-coated on the CF electrode (11 mm diameter) base twice. The resulting electrode was then immersed in a 1 mM solution of the desired monomer in [0.1 M KNO_3_ + 0.1 M PBS] (pH 7.0), subjected to 40 cycles between −0.5 and 1.3 V at 50 mV s^–1^ for the deposition of electropolymerized films, and drop-cast 3 times with 80 μL of [24 mg mL^–1^ FDH + 0.5 wt % PEGDGE] solution in 0.1 M PBS (pH 7.0).

### CF/CNT/SBR/TX/PQ/FDH Bioanodes

An aqueous CNT dispersion (180 μL) containing the dispersant (TX) and binder (SBR) (CNT/SBR/0.5% TX solution = 1 mg:0.53 mg:0.5 mL) was drop-coated on the CF electrode base twice, followed by a single drop-coating of 50 mM PQ/DMF solution (180 μL). The resulting electrode was then drop-cast 3 times with 80 μL of [24 mg mL^–1^ FDH + 0.5 wt % PEGDGE] solution in 0.1 M PBS (pH 7.0).

### CF/SCR/PGluNa/DI/(polyMB, polyTH, or polyAA)/FDH Bioanodes

CF was hydrophilized by 10 min of UV-ozone pretreatment (SSP16–110, Sen Light) prior to use. SCR was prepared by carbonizing sucrose at 500–1200 °C for 1 h under Ar. An aqueous SCR dispersion (180 μL) containing PGluNa as a binder (SCR/1 wt % PGluNa solution = 1 mg:0.5 mL) was drop-coated on the CF electrode base twice. The resulting electrode was then immersed into a 1 mM solution of the desired monomer in [0.1 M KNO_3_ + 0.1 M PBS] (pH 7.0), subjected to 40 cycles between −0.5 and 1.3 V at 50 mV s^–1^ to induce the deposition of electropolymerized films, and drop-coated 3 times with 80 μL of [24 mg mL^–1^ FDH + 0.5 wt % PEGDGE] solution in 0.1 M PBS (pH 7.0).

### CP/PGluNa/Nafion/KB/ABTS/BOD Gas-Diffusion Biocathodes

The electrode slurry was prepared by thoroughly mixing KB powder, ABTS, 5 wt % Nafion dispersion, and 1 wt % PGluNa solution (KB/ABTS/Nafion/binder mass ratio = 60:20:10:15) in a mortar. The CP sheet (4 cm × 4 cm, TGP-H-120, Toray) used as the electrode substrate was hydrophilized by 10 min of UV-ozone pretreatment (SSP16–110, Sen Light), spray-coated with the slurry (KB loading = 30 mg) on one side using an air brush (PS-290, GSI Creos), dried at 80 °C overnight, and hydrophilized as above. A solution of BOD (1200 μL, 25 mg mL^–1^) in 0.1 M PBS (pH 7.0) was drop-cast onto the KB-coated CP sheet (2.4 cm × 2.4 cm), and the obtained electrode was dried at room temperature (∼25 °C) under reduced pressure.

### Measurements

The morphology of the bioanode surface was observed by scanning electron microscopy (SEM; JCM-6000, JEOL). The performance of CF-based bioanodes was evaluated by cyclic voltammetry using a potentiostat (HZ-3000, Hokuto Denko Corporation, Japan) in a three-electrode cell configuration comprising the working electrode (bioanode), counter electrode (Pt wire), and reference electrode (Ag/AgCl in saturated aqueous KCl).

## Results and Discussion

### Electropolymerization and Catalysis Behaviors of MB, TH, and AA

**Figure 1:**
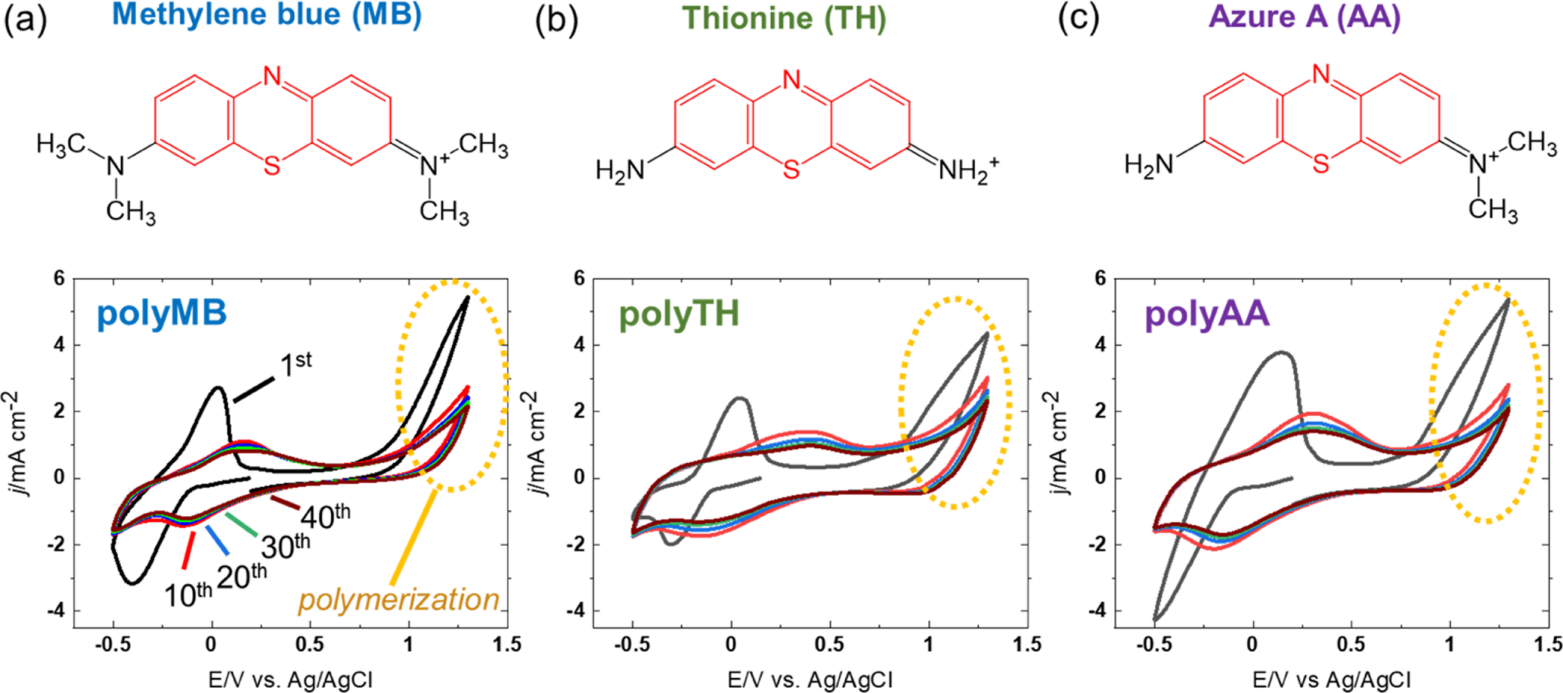
Cyclic voltammograms recorded for the electropolymerization of (a) methylene blue (MB), (b) thionine (TH), and (c) Azure A (AA) at 50 mV s^–1^ in 0.1 M phosphate buffer solution (pH 7.0) with 0.1 M KNO_3_ and 1 mM monomer. The phenothiazine group is highlighted in red.

Figure 1 shows the structures and cyclic voltammograms of the employed phenothiazine derivatives (MB, TH, and AA, respectively). For all dyes, the oxidation peak initially appeared at ∼0 V (vs Ag/AgCl) and shifted in the positive direction during cycling. The initially observed (first-cycle) peak was assigned to the redox reactions of the monomers, whereas the positively shifted peak observed after the 10th cycle corresponded to the redox reactions of polymeric species anodically deposited above 0.8 V.^54−59^ The large oxidation currents obtained at 1.0–1.3 V (vs Ag/AgCl) for each dye indicated that the monomers were oxidized in the high-potential region and that electropolymerization was repeated to form the polymer layer, typically for several tens of cycles.^56^

**Table 1:** Parameters for CV Measurements Conducted in This Study

figure #	enzyme	coenzyme	substrate	catalyst	carbon	binder	surfactant	current density (mA cm^–2^)
2e			NADH		CNT	SBR	TX	(high overpotential)
2f			NADH	polyMB	CNT	SBR	TX	0.22
2g			NADH	polyTH	CNT	SBR	TX	0.32
2h			NADH	polyAA	CNT	SBR	TX	0.27
3	FDH	NAD	formate	polyMB	CNT	SBR	TX	1.09
3	FDH	NAD	formate	polyTH	CNT	SBR	TX	0.89
3	FDH	NAD	formate	polyAA	CNT	SBR	TX	0.91
5ad	FDH	NAD	formate	polyMB	CNT	SBR	TX	1.09
5ab	FDH	NAD	formate	polyMB	SCR-900	SBR	TX	0.93
5bc	FDH	NAD	formate	polyMB	SCR-900	SBR		0.87
5cd	FDH	NAD	formate	polyMB	SCR-900	PGluNa		1.06

**Figure 2:**
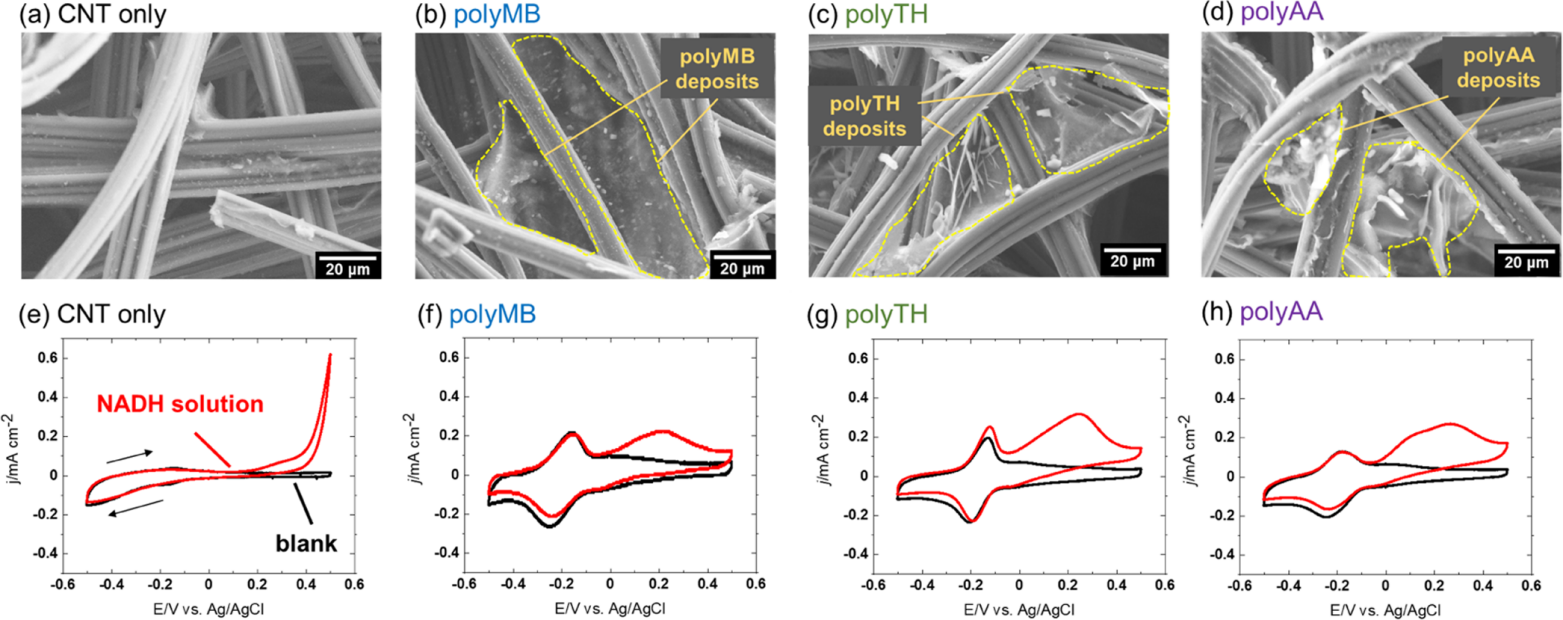
SEM images and cyclic voltammograms of (a, e) CNT/SBR/TX-, (b, f) CNT/SBR/TX/polyMB-, (c, g) CNT/SBR/TX/polyTH-, and (d, h) CNT/SBR/TX/polyAA-modified electrodes recorded at 2 mV s^–1^ in 0.1 M PBS with (red lines) and without (black lines) 10 mM NADH in the absence of fuel. PolyMB, polyTH, and polyAA were electrodeposited for 40 cycles.

SEM imaging (Figure 2a–d) showed that only the smooth surface of carbon fibers was observed on the surface of the electrode modified only with the CNT dispersion (Figure 2a), whereas membrane-like structures were observed for dye-modified electrodes (Figure 2b–d), indicating that CF was successfully modified with the polymeric electrocatalysts. In addition, the monomer-dye identity had no influence on the morphologies of the correspondingly modified electrodes.

The ability of electropolymerized films to catalyze the oxidation of NADH was probed by cyclic voltammetry measurements in 0.1 M PBS containing 10 mM NADH (Figure 2e–h and Table 1). The potential was first scanned in the negative direction and cycled in the range of −0.5 to 0.5 V vs Ag/AgCl. The redox peaks around −0.2 V in Figure 2f–h were not observed in Figure 2e and corresponded to the P-type redox reactions of polyMB, polyTH, and polyAA, respectively. The oxidized form of phenothiazine derivative is capable of oxidizing NADH,^61^ leading to an electrocatalytic mediation reaction. In addition, the larger double-layer capacitance in Figure 2f–h compared to that in Figure 2e possibly indicated the pseudocapacitance of the electropolymerized films. Figure 2e shows that the oxidation current increased in the NADH-containing solution above ∼0.2 V. This anodic current was attributed to the oxidation of NADH to NAD and indicated that the oxidation of NADH occurs at ∼0.2 V without catalyst modification. In contrast, for electrodes modified with electrocatalyst polymers, the oxidation current was observed to be around −0.05 V (Figure 2f–h). These results confirmed that the electrodeposited polymer on the electrode acted as an electrocatalyst to reduce the overpotential of NADH oxidation.

Subsequently, the electrocatalyst polymer–modified electrodes were covered with the enzyme via drop-casting and evaluated by cyclic voltammetry in 0.1 M PBS containing 0.15 M sodium formate and 10 mM NAD. First, the effect of the cross-linking agent used for enzyme modification was examined. Figure S1 shows the cyclic voltammograms of electrodes prepared using GA^62^ and PEGDGE^18^ as enzyme cross-linkers and polyMB as the electrocatalyst. PolyMB was selected based on the previous report.^44^ The redox couple observed around −0.2 V was ascribed to the redox reactions of polyMB, as also shown in Figure 2f. In Figure S1a, the blank curve almost overlaps with the curve obtained in the fuel solution, while remarkable fuel oxidation can be observed starting from −0.05 V in Figure S1b. This oxidation potential is consistent with that observed in Figure 2f for NADH oxidation at the polyMB-modified electrode, indicating the oxidation of NADH formed through the reduction of NAD, which proceeds simultaneously with FDH-catalyzed formate oxidation. GA-mediated cross-linking is a dehydration-condensation process known as a strong cross-linking reaction and is capable of causing enzymatic activity loss. In contrast, PEGDGE cross-links via epoxy-ring-opening and is a relatively mild agent enabling electrode fabrication without enzymatic activity loss.^63^ Therefore, PEGDGE was selected as the enzymatic cross-linker.

Second, for PEGDGE loading optimization, we prepared electrodes using cross-linker loadings of 0, 0.5, 1.0, 1.5, and 2.0 wt %. Figure S2 shows that the current density was maximized at PEGDGE loadings of 0.5 and 1.0 wt %, as larger loadings caused conformational changes in the enzyme.^64^ In the absence of the cross-linker, a large deviation of the maximum current density was observed, as the physically absorbed enzyme was not stably immobilized on the electrode. Therefore, a loading of 0.5 wt % PEGDGE providing a stable and higher current density was selected for further experiments.

**Figure 3:**
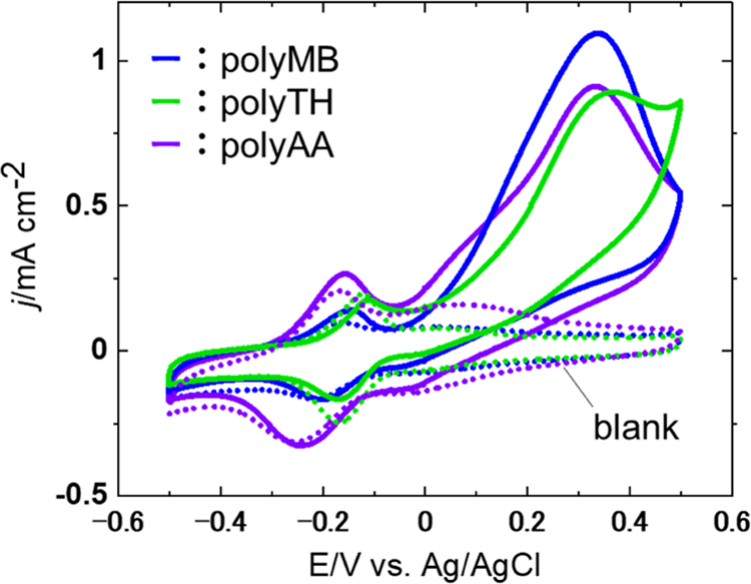
Cyclic voltammograms of CNT/SBR/TX/polyMB/FDH-, CNT/SBR/TX/polyTH/FDH-, and CNT/SBR/TX/polyAA/FDH-modified electrodes recorded at 2 mV s^–1^ in [0.1 M PBS + 10 mM NAD] with (solid lines) and without (dashed lines) 0.15 M sodium formate.

Figure 3 presents the cyclic voltammograms of electrodes modified with different electropolymerized dyes at an optimized PEGDGE addition of 0.5 wt %. The redox peaks observed around −0.2 V indicated the reversible redox of electropolymerized films, as mentioned in Figure S1. For all electrodes, an increase in current density was observed beyond −0.05 V in the presence of fuel, in agreement with NADH oxidation shown in Figure 2f–h. Thus, this oxidation current was assigned to the oxidation of NADH formed by the reduction of NAD, which proceeded simultaneously with FDH-catalyzed formate oxidation. The larger oxidation currents in Figure 3 compared with those in Figure 2f–h indicate that the mediated formate oxidation proceeded in the presence of FDH and NAD. The maximum current densities obtained for polyMB, polyTH, and polyAA were 1.09, 0.89, and 0.91 mA cm^–2^, respectively. Figure S3 displays the reproducibility test data of the three electrodes, revealing that the highest and most reproducible maximum current density was achieved in the case of polyMB. Given the difficulty of revealing the structure–property relationships of electrocatalysts and enzymes/coenzymes due to the complicated structure of enzymes, as described in previous works also used exhaustive trials.^65−67^ The results of rotating disk electrode experiments (Figure S4) and Kouteck–Levich fitting (Figure S5) also indicated that the highest kinetic current was observed for polyMB. Thus, polyMB was concluded to exhibit the highest catalytic activity and reproducibility.

The electrochemical properties of formic acid bioanodes were also investigated in the presence of quinones, which are typical mediators for NAD-dependent bioanodes.^26,43^Figure S6 shows the cyclic voltammogram of a formate bioanode with PQ as the electrocatalyst. The oxidation peak at around 0 V and the reduction peak at around −0.3 V, which appeared in both the presence and absence of fuel, originated from the redox reactions of PQ. An increase in current density starting from ∼0.2 V indicated the oxidation of formate by FDH and the simultaneous oxidation of NADH (Figure 3). However, the maximum current density (195 μA cm^–2^ at 0.5 V in Figure S6) was significantly lower than that observed for polyMB as the electrocatalyst.

### Fabrication of HCOO^–^/O_2_ Biofuel Cell with Surfactant-Free Bioanode and Gas-Diffusion Biocathode

**Figure 4:**
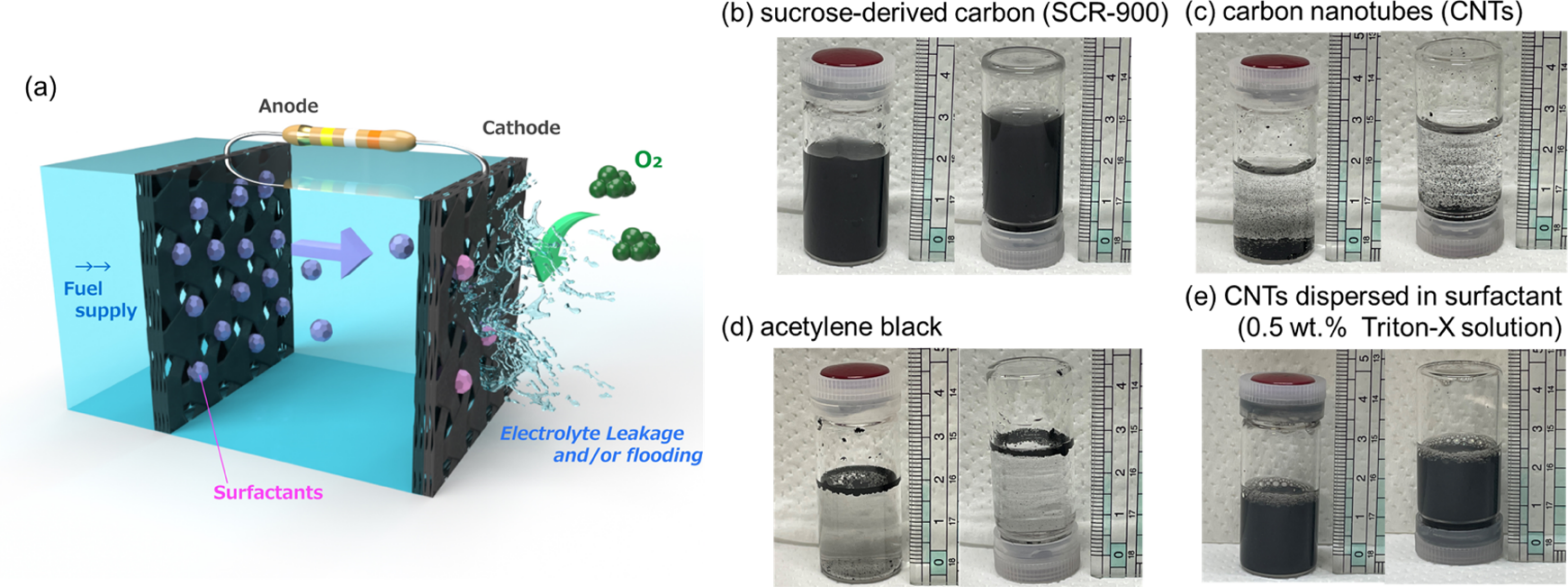
(a) Schematic illustration of the full cell composed of the surfactant-containing bioanode and gas-diffusion biocathode (see the photograph in Figure S7) and dispersibility of (b) SCR-900, (c) CNTs, (d) acetylene black, and (e) CNTs with TX in water.

Based on the above results, the CF/CNT/SBR/TX/polyMB/FDH bioanode was found to be optimal and applied to a full cell in combination with a gas-diffusion-type biocathode. A schematic illustration and photograph of the fabricated full cell are shown in Figures 4a and S7. After cell assembly, the electrolyte solution slowly leaked into the gas compartment. We found that the leakage was caused by the gradual dissolution of the surfactant (TX) from the CF/CNT/SBR/TX/polyMB/FDH bioanode in the electrolyte and the resulting excessive hydrophilization of the opposed gas-diffusion biocathode inside the full cell. Given the importance of appropriate hydrophobicity for gas-diffusion biocathodes,^49,62,68^ surfactant-free bioanodes are required to design optimal full cells. Therefore, based on our previous study, a water-dispersible carbon material was synthesized by heat-treating sucrose at 900 °C for 1 h under Ar.^60^Figure 4b–e shows that SCR-900 (SCR obtained at 900 °C) was well dispersible in water. CNTs also showed good dispersibility in the presence of TX, whereas hydrophobic CNTs and acetylene black were not dispersible in water in the absence of a surfactant. This behavior indicated that unlike typical carbon materials such as CNT and acetylene black, SCR-900 did not require a surfactant for dispersion. The high dispersibility of SCR-900 could have originated from its relatively low synthesis temperature, which preserved surface functional groups capable of interacting with water.

**Figure 5:**
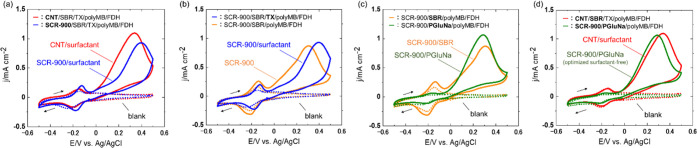
Cyclic voltammograms recorded in 0.1 M PBS with (solid lines) and without (dashed lines) 0.15 M sodium formate and 10 mM NAD at 2 mV s^–1^ for (a) CNT/SBR/TX/polyMB/FDH- and SCR-900/SBR/TX/polyMB/FDH-modified electrodes, (b) SCR-900/SBR/TX/polyMB/FDH- and SCR-900/SBR/polyMB/FDH-modified electrodes, (c) SCR-900/SBR/polyMB/FDH- and SCR-900/PGluNa/polyMB/FDH-modified electrodes, and (d) CNT/SBR/TX/polyMB/FDH- and SCR-900/PGluNa/polyMB/FDH-modified electrodes. Reproducibility data can be found in Figure S8.

Figure 5a presents the cyclic voltammograms of CNT/SBR/TX/polyMB/FDH- and SCR-900/SBR/TX/polyMB/FDH-modified electrodes recorded in a solution containing 0.1 M PBS, 0.15 M sodium formate, and 10 mM NAD. The oxidation current derived from formate oxidation was observed around −0.05 V for both electrodes and was similar to the oxidation currents in Figures S1 and 3, which indicated the occurrence of a mediation reaction at the electrodes. The maximum current densities of CNT/surfactant and SCR-900/surfactant electrodes equaled 1.09 and 0.93 mA cm^–2^, respectively; i.e., SCR-900/surfactant exhibited a performance similar to that of CNT/surfactant as an enzyme support. The slight inferiority of the former electrode was ascribed to the lower electronic conductivity of SCR due to its low synthesis temperature and lower surface area compared to those of CNTs. Surfactant-free bioanodes were prepared using an aqueous dispersion of SCR-900. Figure 5b shows the voltammograms obtained for surfactant-free and surfactant-containing SCR-900/SBR/polyMB/FDH-modified electrodes, revealing that the maximum current densities were slightly lower in the former case. To further improve the electrode performance, we replaced SBR latex binder with the more strongly bindable PGluNa^18,69^ (Figure 5c). The SCR-900/PGluNa/polyMB/FDH-modified electrode showed a higher maximum current density (1.06 mA cm^–2^) than the SBR-based electrode, which was due to the formation of an electrode surface more favorable for enzyme and electrocatalyst immobilization because of the improved adhesion properties of PGluNa. The optimized surfactant-free electrode (SCR-900/PGluNa/polyMB/FDH) and the CNT/surfactant electrode (CNT/SBR/TX/polyMB/FDH) achieved comparable current densities (1.06 and 1.09 mA cm^–2^, respectively; Figure 5d), which evidenced that SCR can be an alternative to CNTs as a carbon support and that surfactant-free bioanodes were successfully fabricated.

Figures S9 and S10 show that with increasing SCR synthesis temperature (500–1200 °C), current density increased, maximized at 900 °C, and decreased for 1000, 1100, and 1200 °C SCRs. With increasing synthesis temperature (carbonization degree), electronic conductivity increased, whereas the number of surface functional groups decreased, resulting in a decrease in dispersibility in aqueous media. By considering the trade-off between these factors, we found the optimal bioanode performance was observed by using sucrose-derived carbon synthesized at 900 °C.^60^

**Figure 6:**
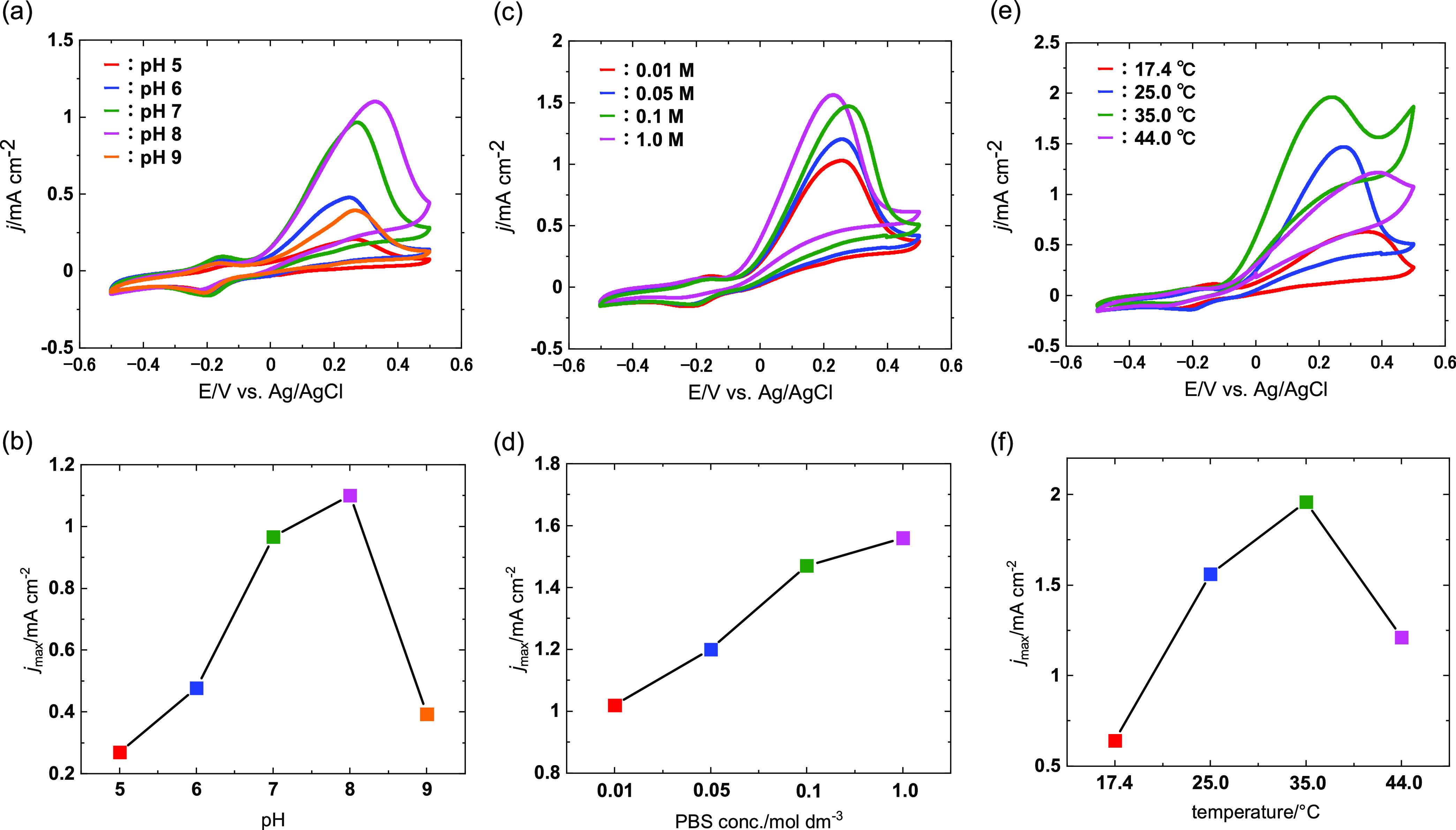
Cyclic voltammograms of SCR-900/PGluNa/polyMB/FDH electrodes recorded at 2 mV s^–1^ at various (a, b) pH, (c, d) PBS concentrations, and (e, f) temperatures. All electrolytes contained 0.15 M sodium formate and 10 mM NAD; 0.1 M PBS was used for (a, b), pH 8 was used for (b–f), and 1.0 M PBS was used for (e, f). Measurements were conducted at 25 °C in (a–d).

Figure 6 further presents the results of operation condition optimization. In the case of pH optimization, cyclic voltammograms were recorded for the SCR-900/PGluNa/polyMB/FDH-modified electrode in PBSs at pH 5, 6, 7, 8, and 9 (Figure 6a,b). Current density increased with an increase in pH from 5 to 8 and rapidly decreased upon a further pH increase to 9, which agreed with the optimum pH for FDH (∼8). In acidic conditions, this alteration is linked to the ionization of two carboxy groups present at the NAD-binding site within the enzyme’s active center. Conversely, a shift to alkaline pH prompts a structural transformation, influencing the enzyme’s substrate affinity.^70^ Therefore, PBS of pH = 8 was selected for subsequent experiments. Figure 6c,d demonstrates the dependence of PBS concentration under pH 8 conditions, showing that the highest current density of 1.56 mA cm^–2^ was obtained in 1.0 M PBS. Thus, the measurement temperature was optimized in 1.0 M PBS at pH 8.0 (Figure 6e,f). With elevating temperature from 17 to 44 °C, the peak current density increased at 35 °C (1.96 mA cm^–2^) and decreased upon a further temperature increase to 44 °C (1.21 mA cm^–2^). This volcano-type relationship suggested a trade-off between the low reaction rate in the low-temperature region and the partial degradation of the enzyme steric structure at higher temperatures. The optimum temperature aligns with the previously reported value for free *C. boidinii* FDH (37 °C).^71^ This suggests that the enzyme immobilization technique employed in this study is suitable. It is noteworthy that optimum temperatures for immobilized enzymes can vary significantly based on the immobilization method used. Thus, the optimal operation conditions for the formate bioanode using NAD-dependent FDH were identified as 1.0 M PBS with pH 8.0 and 35 °C.

Finally, a full cell was fabricated by combining an SCR-900/PGluNa/polyMB/FDH bioanode and a CP/PGluNa/Nafion/KB/ABTS/BOD gas-diffusion-type biocathode. Note that the full cell was operated under the more practical (although nonoptimal) conditions of 0.1 M PBS (pH 8) and 25 °C. In a preliminary test, cyclic voltammetry for the cathode side was performed in 0.1 M PBS containing 0.15 M sodium formate and 10 mM NAD to evaluate the CP/PGluNa/Nafion/KB/ABTS/BOD gas-diffusion biocathode (Figure S11). Current derived from oxygen reduction was successfully obtained starting from ∼0.5 V vs Ag/AgCl, with a maximum current density of −1.13 mA cm^–2^ observed around 0.3 V.

**Figure 7:**
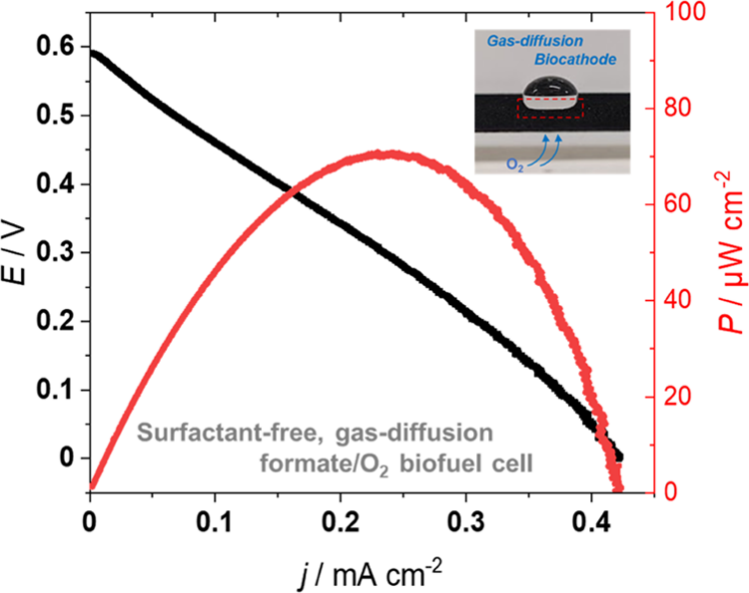
*I*–*V* curve of the formate/O_2_ biofuel cell with a surfactant-free bioanode operated at room temperature (∼25 °C). Anode: SCR-900/PGluNa/polyMB/FDH, cathode: CP/PGluNa/Nafion/KB/ABTS/BOD gas-diffusion biocathode; electrolyte: 0.1 M PBS with 0.15 M sodium formate and 10 mM NAD (pH 8). Cell current was measured by linear sweep voltammetry at 1 mV s^–1^. The inset photograph is the gas-diffusion biocathode, showing an example of an electrolyte/gas phase interface.

Images of the fabricated full cell are shown in Figure S12. The elimination of the surfactant from the bioanode can successfully avoid the electrolyte leaking across the BOD-modified CP into the gas phase compartment and allow for successful full cell fabrication. The *I*–*V* curve of the full cell obtained through linear sweep voltammetry at 1 mV s^–1^ is shown in Figure 7, and the corresponding open-circuit voltage, maximum current density, and maximum power density were obtained as 0.59 V, 0.42 mA cm^–2^, and 70 μW cm^–2^, respectively, based on the geometric area of bioanode. In this case, current density was limited by the bioanode side, as the electrode size for the bioanode (11 mm diameter) is smaller than the biocathode (2.4 cm × 2.4 cm), while the performance for each electrode is nearly identical (1.56 mA cm^–2^ for the bioanode and 1.13 mA cm^–2^ for biocathode). Although the fabricated cell was expected to deliver a higher electromotive force, its open-circuit voltage was comparable to that of a previously reported glucose/O_2_ biofuel cell (0.62 V),^49^ which was ascribed to the fact that the electrocatalyst used in this study (polyMB) had a relatively high NADH oxidation potential. The search for appropriate electrocatalysts is therefore further required to achieve high-voltage and high-power formate-based biofuel cells.

## Conclusions

A formate bioanode utilizing a NAD-dependent formate dehydrogenase was fabricated by using an electropolymerized film of methylene blue (polyMB) and highly water-dispersible carbon materials obtained by annealing sucrose at 900 °C (SCR-900). Owing to the high catalytic activity of polyMB for NADH oxidation and the use of SCR-900, the bioanode achieved a current density of 1.96 mA cm^–2^ and was devoid of any surfactant dissolution. The full cell fabricated using SCR-900 as a carbon support for the bioanode side maintained an appropriately hydrophobic biocathode side because of the absence of surfactants and delivered an open-circuit voltage of 0.59 V and a maximum power density of 70 μW cm^–2^. Thus, this study demonstrates the effectiveness of formic acid as a fuel for biofuel cells and suggests new possibilities for the application of carbon materials in bioanodes.

## Supporting Information Available

CVs with different cross-linker (Figures S1 and S2); reproducibility of Figure 3 (Figure S3); RDE results (Figures S4 and S5); CVs with different redox mediator (Figure S6); schematic diagram of full cell with surfactant (Figure S7); reproducibility of Figure 5 (Figure S8); CV with different SCR synthesis temperatures (Figures S9 and S10); CV of biocathode (Figure S11); and schematic diagram of full cell without surfactant (Figure S12) (PDF)

## Abbreviations

NAD: nicotinamide adenine dinucleotide
CNT: carbon nanotube
SCR: sucrose-derived carbon
FDH: formate dehydrogenase
BOD: bilirubin oxidase
PQ: 9,10-phenanthrenequinone
MB: methylene blue
TH: thionin acetate
AA: Azure A chloride
DMF: dimethylformamide
ABTS: 2,2′-azino-bis(3-ethylbenzothiazoline-6-sulfonic acid)
GA: glutaraldehyde
PEGDGE: poly(ethylene glycol) diglycidyl ether
TX: Triton X-100
PGluNa: sodium poly-γ-glutamate
SBR: styrene–butadiene rubber
KB: Ketjen black
AB: acetylene black
CF: carbon fabric
CP: carbon paper
PBS: phosphate-buffered solutions
DI: deionized water
GCE: glassy carbon electrode
