# Supplementary material for: Surfactant-Free Formate/O2 Biofuel Cell with Electropolymerized Phenothiazine Derivative-Modified Enzymatic Bioanode
Source: ACS Appl Bio Mater. 2023 Sep 26;6(10):4304–13. doi: 10.1021/acsabm.3c00502 (PMC10583231; doi:10.1021/acsabm.3c00502)
Supplement: Supplementary file 1 — mt3c00502_si_001.pdf [file mt3c00502_si_001.pdf]

Supporting Information

Surfactant–Free Formate/O_2 Biofuel Cell with Electropolymerized Phenothiazine Derivative–Modified Enzymatic Bioanode

Motohiro Kosugi, Ryoichi Tatara, Yuki Fujii and Shinichi Komaba*

Department of Applied Chemistry, Tokyo University of Science, Shinjuku, Tokyo 162-8601, Japan

*Corresponding author. Email: komaba@rs.tus.ac.jp

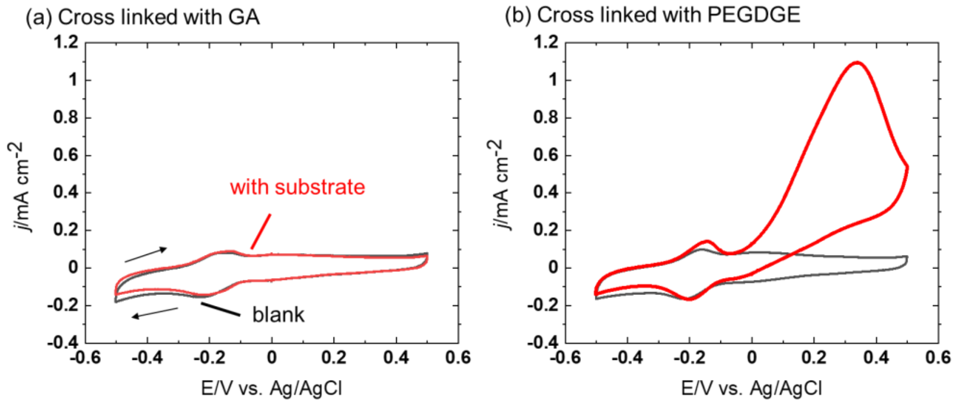

Figure S1. Cyclic voltammograms of (a) CNT/SBR/TX/polyMB/FDH (crosslinked with 0.5 wt% GA)- and (b) CNT/SBR/TX/polyMB/FDH (crosslinked with 0.5 wt% PEGDGE)-modified electrodes recorded at 2 mV s^{-1} in 0.1 M PBS + 10 mM NAD with (red lines) and without (black lines) 0.15 M sodium formate.

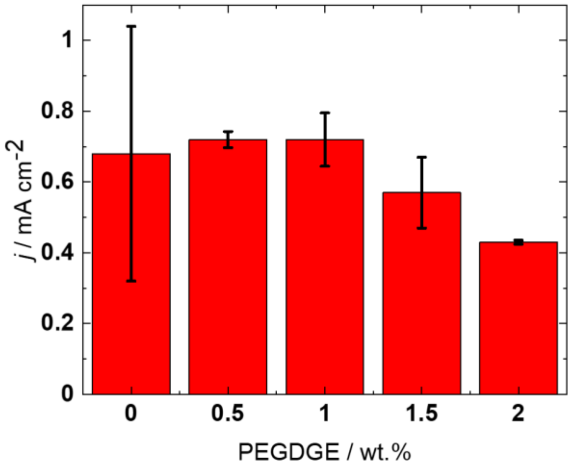

Figure S2. Maximum current density achieved by CNT/SBR/TX/polyMB/FDH (crosslinked with PEGDGE)-modified electrodes at 2 mV s^{-1} in 0.1 M PBS with 0.15 M sodium formate and 10 mM NAD. The results indicate averages and standard deviations (n = 3).

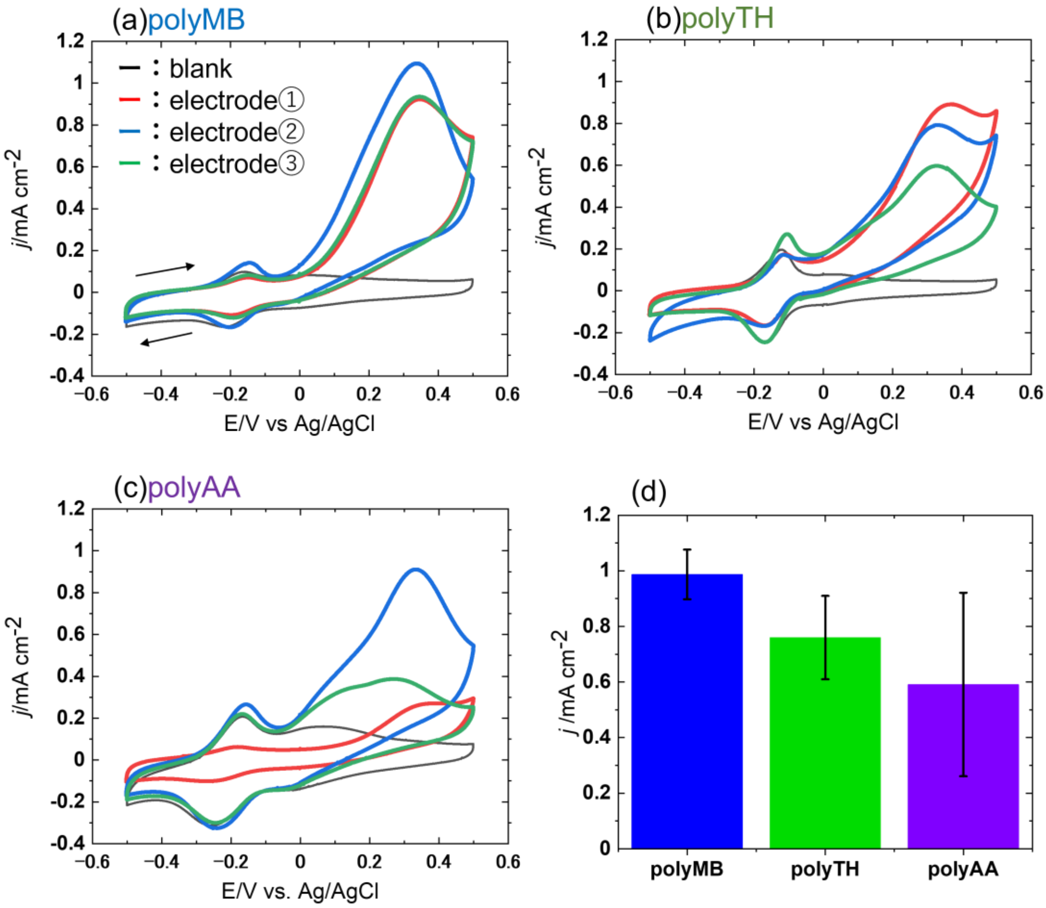

Figure S3. Reproducibility of formate bioanodes fabricated using (a) polyMB, (b) polyTH, and (c) polyAA (see **Figure 3**). The averages and deviations of the observed current densities are shown in (d). Data were measured for CNT/SBR/TX/[poly(MB), poly(TH), or poly(AA)]/FDH-modified electrodes at 2 mV s^{-1} in 0.1 M PBS + 10 mM NAD with and without (blank) 0.15 M sodium formate.

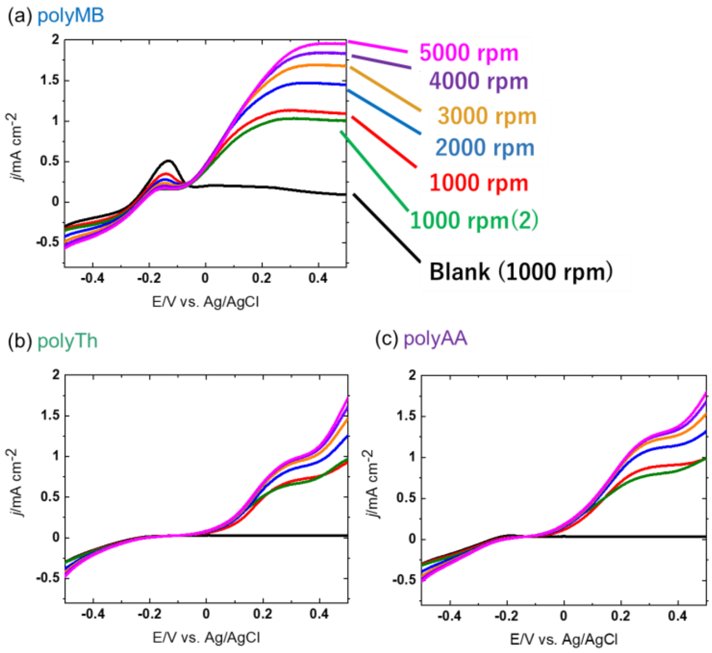

Figure S4. Hydrodynamic voltammograms of (a) CNT/SBR/TX/polyMB-, (b) CNT/SBR/TX/polyTH- and (c) CNT/SBR/TX/polyAA-modified electrodes recorded at 2 mV s^{-1} in 0.1 M PBS with 10 mM NADH and without any fuel (blank). The experiments were conducted in the following order: 1000, 2000, 3000, 4000, and 5000 rpm, and then back to 1000 rpm (2).

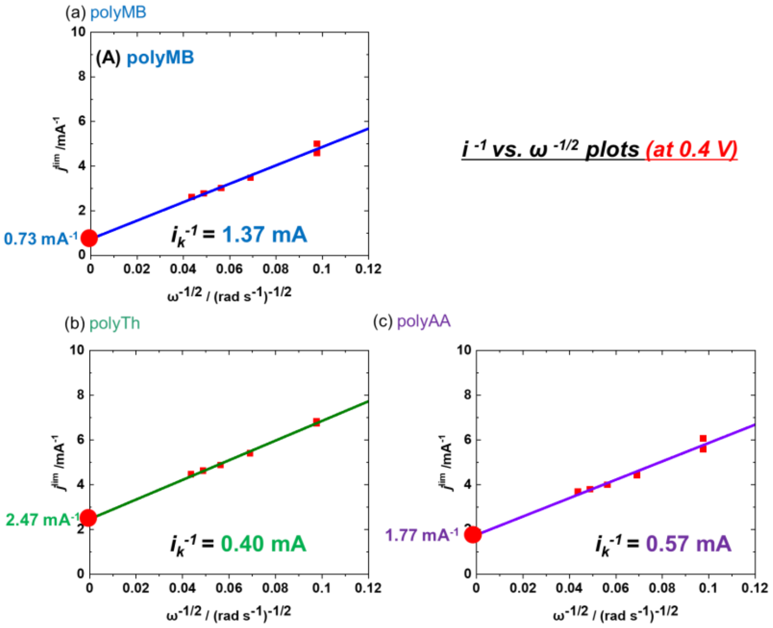

Figure S5. Koutecky-Levich plots obtained for (a) CNT/SBR/TX/polyMB-, (b) CNT/SBR/TX/polyTH- and (c) CNT/SBR/TX/polyAA-modified electrodes at 2 mV s^{-1} in 0.1 M PBS with 10 mM NADH.

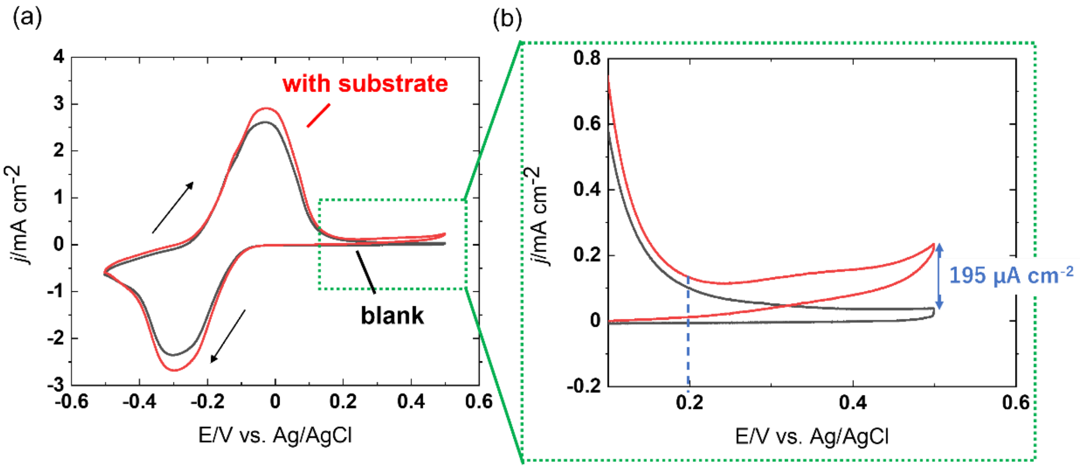

Figure S6. Cyclic voltammograms of CNT/SBR/TX/PQ/FDH-modified electrodes recorded at 2 mV s^{-1} in 0.1 M PBS with (red) and without (black) 0.15 M sodium formate and 10 mM NAD in the ranges of (a) −0.6 to 0.6 V and (b) 0.1 to 0.6 V.

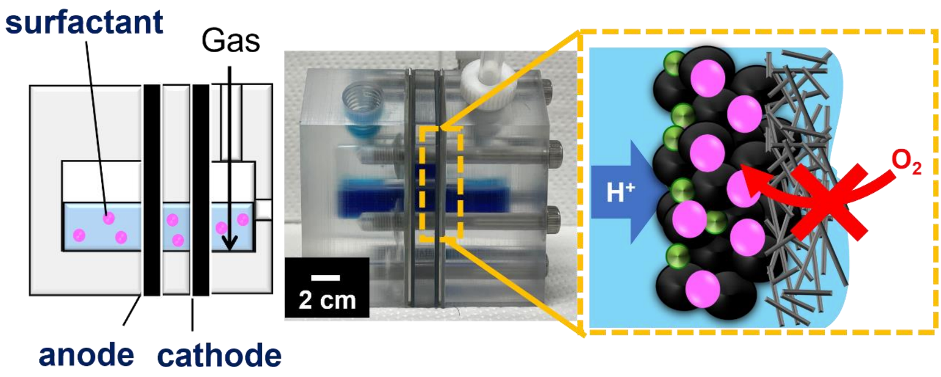

Figure S7. Schematic diagram and image of the full cell with the CNT/SBR/TX/polyMB/FDH bioanode and gas-diffusion biocathode. For clarity, the electrolyte was colored using a dye.

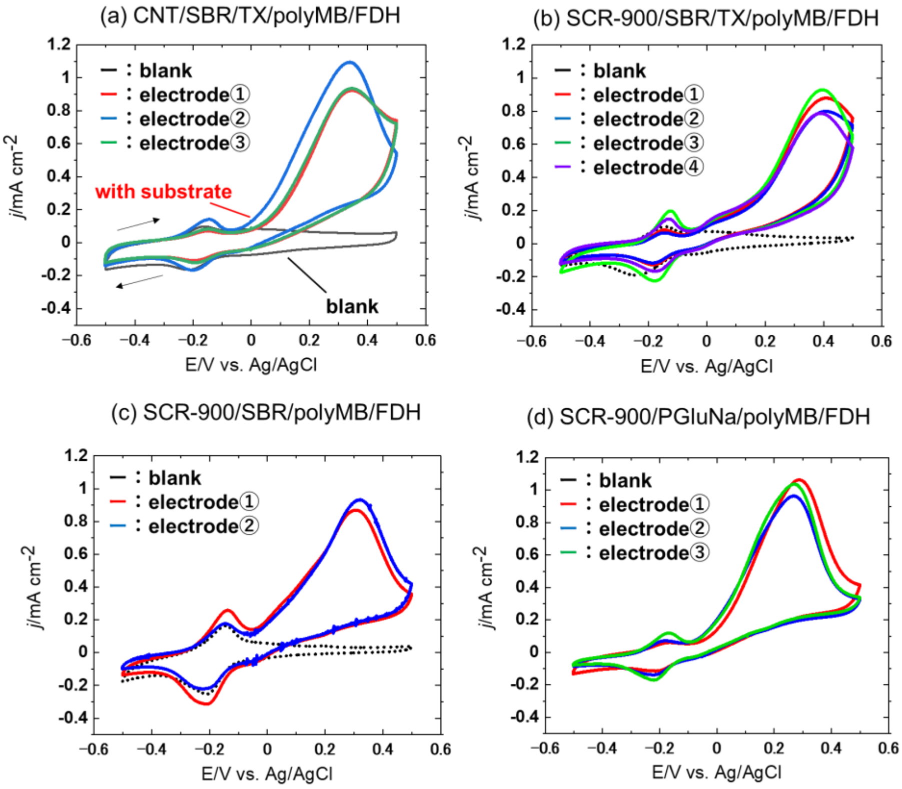

Figure S8. Reproducibility of the fabricated formate bioanodes (see **Figure 5**). Cyclic voltammograms were recorded in 0.1 M PBS with 0.15 M sodium formate and 10 mM NAD at 2 mV s^{-1}. (a) CNT/SBR/TX/polyMB/FDH-, (b)SCR-900/SBR/TX/polyMB/FDH-modified electrodes, (c) SCR-900/SBR/polyMB/FDH-modified electrodes, and (d) SCR-900/PGluNa/polyMB/FDH-modified electrodes.

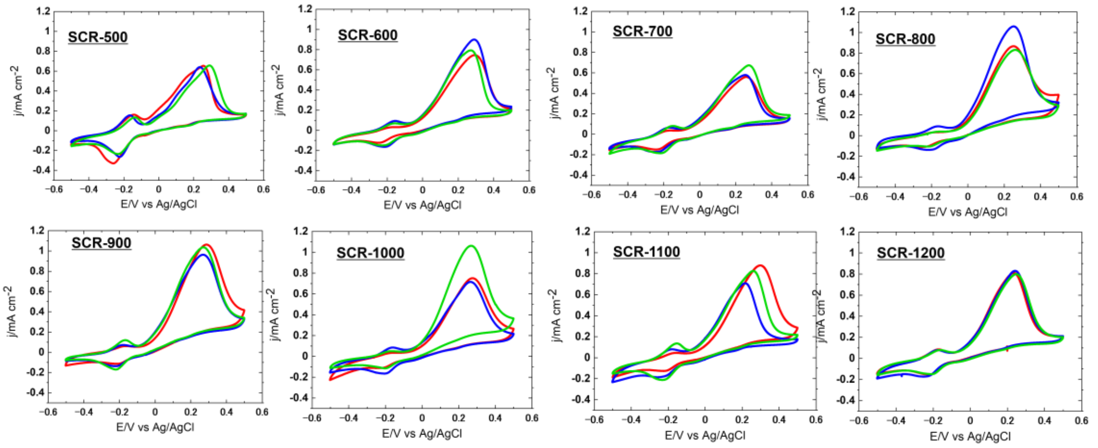

Figure S9. Effects of the SCR synthesis temperature on the cyclic voltammograms of SCR/PGluNa/polyMB/FDH-modified electrodes recorded in 0.1 M PBS with 0.15 M sodium formate and 10 mM NAD at 2 mV s^{-1}. The color variation indicates reproducibility, providing the average and deviation as summarized in **Figure S10**.

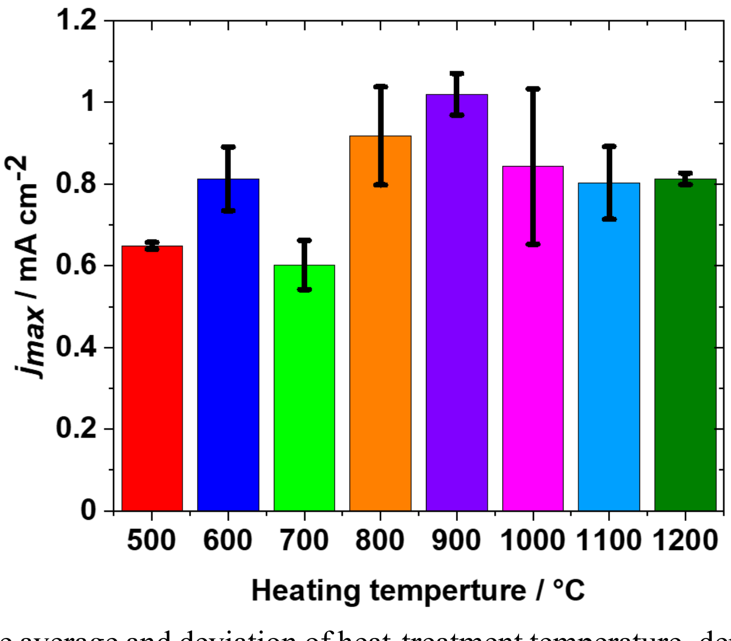

Figure S10. The average and deviation of heat-treatment temperature–dependent formate bioanode performances shown in **Figure S9**.

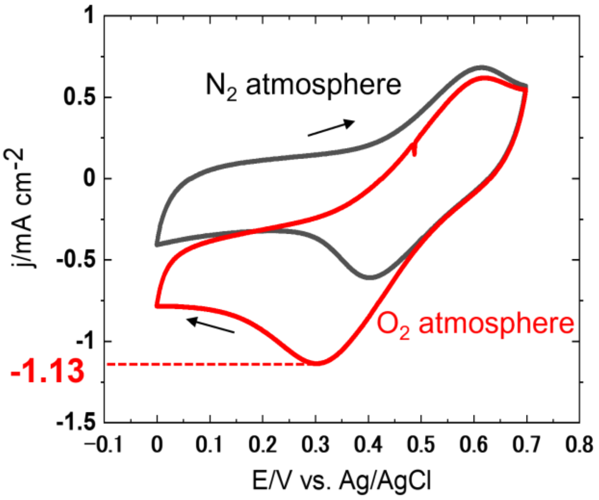

Figure S11. Cyclic voltammograms of CP/PGluNa/Nafion/KB/ABTS/BOD gas-diffusion biocathode recorded under O_2 and N_2 at 2 mV s^{-1} in 0.1 M PBS with 0.15 M sodium formate and 10 mM NAD.

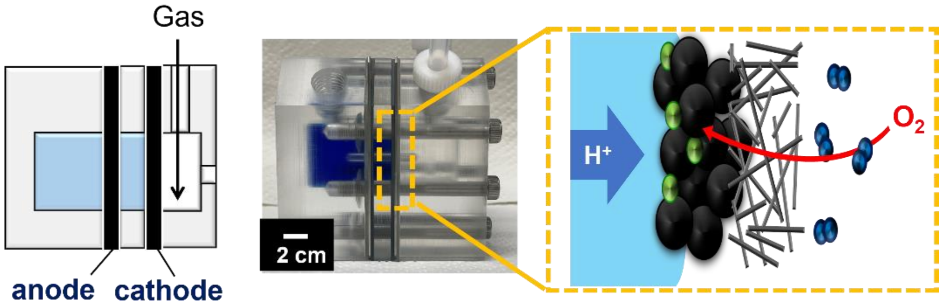

Figure S12. Schematic diagram and image of the full cell with the SCR-900/PGluNa/polyMB/FDH-modified bioanode and the CP/PGluNa/Nafion/KB/ABTS/BOD gas-diffusion-type biocathode. For clarity, the electrolyte was colored using a dye.
